# Effects of Cytokinin and Nitrogen on Drought Tolerance of Creeping Bentgrass

**DOI:** 10.1371/journal.pone.0154005

**Published:** 2016-04-21

**Authors:** Zhihui Chang, Yang Liu, Hui Dong, Ke Teng, Liebao Han, Xunzhong Zhang

**Affiliations:** 1 College of Forestry, Beijing Forestry University, Beijing, 100083, P.R. China; 2 Department of Crop and Soil Environmental Sciences, Virginia Polytechnic Institute and State University, Blacksburg, Virginia, 24061, United States of America; National Taiwan University, TAIWAN

## Abstract

Cytokinin (CK) is a vital plant hormone that controls many aspects of growth and development in plants. Nitrogen (N) is the indispensable macronutrient needed in plants and also one of the most important limiting factors for plant growth. This study was designed to investigate the simultaneous effects of CK and N on the visual turf quality and antioxidant metabolism of drought-stressed creeping bentgrass (*Agrostis stolonifera* L.). ‘PennA-4’ creeping bentgrass treated with *trans*-zeatin riboside at three rates of CK concentrations of 0, 10 and 100 μM (designated by CK0, 10, and 100) and two nitrogen rates with 2.5 and 7.5 kg N·ha^-1^ every 15 days (designated by low and high N) in a complete factorial arrangement was grown under two soil moisture regimes: well-watered and drought stress. Exogenous CK improved turf quality and delayed leaf wilting under drought stress, especially under high N. The grasses treated with CK10 and CK100 had lower O_2_^-^ production and H_2_O_2_ concentration than those without CK treatment. The CK100 treatment enhanced the activities of superoxide dismutase (SOD), ascorbate peroxidase (APX), catalase (CAT), and guaiacol peroxidase (POD) by 25%, 22%, 17% and 24%, respectively, relative to CK0. Moreover, the activity changes of the antioxidant enzyme isoforms were more significant under high N condition relative to low N condition. Our results demonstrated the beneficial impacts of CK and N on physiological reactions, especially antioxidant metabolism, and foliar application of CK at 10 or 100 μM plus 7.5 kg ha^-1^ N biweekly may improve drought stress resistance of creeping bentgrass.

## Introduction

Drought stress is one of the most adverse abiotic stresses for turfgrass growth in water-limiting region. Responses of grasses to drought stress include changes in various morphological and physiological elements [1,2]. Drought causes inhibition of the photosynthesis and respiration processes that has been associated with declines in chlorophyll concentration, cell membrane stability, leaf water content and antioxidant enzyme activity [3,4]. Drought stress causes an imbalance of the electron-transfer chain and promotes the production of reactive oxygen species (ROS), including superoxide radical (O_2_^-^), and hydrogen peroxide (H_2_O_2_), which can result in the autocatalytic peroxidation of membrane lipids, causing loss of membrane semipermeability and modification of functionality [5]. Plants have evolved enzymatic system to scavenge ROS [6]. In the enzymatic system, superoxide dismutase (SOD) constitutes the first line of defense against ROS by dismutating the O_2_^-^ to H_2_O_2_. Next, H_2_O_2_ is broken down by catalase (CAT) and an array of peroxidases, such as ascorbate peroxidase (APX) and guaiacol peroxidase (POD) [7]. However, the function of the scavenging enzyme systems can be influenced by drought stress, which can cause increases in lipid peroxidation and consequent membrane damage [8].

Cytokinin (CK) is one of the major hormones that is involved in many different developmental and physiological processes in plants [9,10]. In recent years, many studies provided the evidences that exogenous CK application could improve plant growth, delay leaf senescence, lessen cell membrane lipid peroxidation [11,12] and could improve the tolerance to abiotic stresses in plants [13] including turfgrass [14]. The burst of excess ROS may lead to membrane lipids damage in plant cells [5], while the enzymatic systems are responsible for maintaining the membrane structure by improving the antioxidant capacity against ROS damage [14,15]. Therefore, the stimulation of antioxidant enzymatic systems after the exogenous CK application could be the one of the important protective mechanisms for the plants to avoid abiotic injuries.

Nitrogen (N) is an essential component of many organic compounds in plants, such as proteins, nucleic acids, and some plant hormones [16]. A proper level of N is necessary to sustain regular plant growth as well as to withstand the environmental stresses [17,18,19]. Previous study reported that there was higher cell membrane stability and leaf tissue integrality in soybean (*Glycine max* L.) with higher N level when subjected to drought stress [20]. Researches also showed that the improved heat tolerance of creeping bentgrass (*Agrostis stolonifera* L.) after foliar application of NH_4_NO_3_ could be due to the maintenance of the scavenging ability of antioxidants and inhibition of lipid peroxidation [21,22]. Thus, it seems feasible for us to improve the drought tolerance of creeping bentgrass by manipulating the N concentration and its application method [23,24].

Creeping bentgrass is a primary cool-season turfgrass species used on close-cut turf surfaces such as golf course fairways and putting greens. Many studies indicated that environmental stresses affected physiological changes, especially antioxidant responses, in many cool-season turfgrass species [10,25,26], and further suggested the ability for avoiding oxidative stress is a very important factor in determining the environmental stress tolerance of turfgrasses [22]. In addition, the individual effects of CK or N on the promotion of antioxidant responses have been studied in creeping bentgrass [14,21]. Recently, Wang et al. [22] disclosed the simultaneous effects of exogenous cytokinin and nitrogen on antioxidant metabolism in roots and shoots of creeping bentgrass. However, few studies had been focused on physiological responses and antioxidant metabolism of creeping bentgrass subjected to both exogenous CK and N treatments under drought stress. The objective of this study was to investigate the combined effects of CK and N on the drought tolerance associated with the antioxidant metabolism in creeping bentgrass.

## Materials and Methods

### Plant culture, CK and N application, and drought stress treatment

‘PennA-4’ creeping bentgrass sods with trim height of 4.5 mm were collected from a 10-year-old golf green at Beijing Honghua International Golf Club. The sods were transferred into the square container (20 cm in length, 20 cm width and 25 cm height with holes at the bottom for drainage) filled with a mixture (1/2, v/v) of sand and soil. The soil contained total N at 0.863 g·kg^-1^, NO_3_^—^N at 14.2 mg·kg^-1^, NH_4_^+^-N at 13.0 mg·kg^-1^, total P at 0.618 g·kg^-1^, total K at 24.7 mg·kg^-1^ and organic matter at 16.9 g·kg^-1^. The grasses were fertilized with half-strength N-free Hoagland’s solution [27] and NH_4_NO_3_ at 5 kg N·ha^-1^ every two weeks over the first month. Grasses were cut to 20 mm twice a week during the culture period, except the last week in order for enough sampling for further analysis.

After one month culture, the grasses were transferred into a growth chamber with the growth conditions as following: day/night temperature at 25/15°C, relative humidity at 60%, photosynthetically active radiation at 450 μmol·s^-1^·m^-2^ and day-night photoperiod with 14h/10h. The grasses were sprayed with CK (*trans*-zeatin riboside) and fertilized with NH_4_NO_3_ solution simultaneously. CK was dissolved in trace methanol, and then diluted with deionized water; NH_4_NO_3_ was dissolved in deionized water directly. The treatments included: 1) low N (2.5 kg N·ha^-1^) + CK0 (no *trans*-zeatin riboside); 2) low N (2.5 kg N·ha^-1^) + CK10 (*trans*-zeatin riboside at 10 μM); 3) low N (2.5 kg N·ha^-1^) + CK100 (*trans*-zeatin riboside at 100 μM); 4) high N (7.5 kg N·ha^-1^) + CK0 (no *trans*-zeatin riboside); 5) high N (7.5 kg N·ha^-1^) + CK10 (*trans*-zeatin riboside at 10 μM); 6) high N (7.5 kg N·ha^-1^) + CK100 (*trans*-zeatin riboside at 100 μM). The combined N and CK treatments were applied at 0 d, 15 d and 30 d after moisture treatment. The two rates (10 uM and 100 uM) of *trans*-zeatin riboside were selected based on the results of previous studies by co-author Zhang who found that the two rates were more effective in promoting heat tolerance of creeping bentgrass than other rates (0.1, 1, and 1000 uM; personal communication), and also the results by Liu [27] and Zhang [10].

All grasses were subjected to two soil moisture regimes: well watered and drought stress. The volumetric soil water content of the ‘‘well-watered” containers were maintained at 29% throughout the experiment. For “drought stress” treatment, irrigation was provided to amount of water equivalent to 50% evapotranspiration (ET) during 1–19 d, 40% ET during 20–32 d, and 20–30% ET during 32–40 d. The grasses were watered every 3–4 d and dried down gradually by allowing the volumetric soil water content to drop to about 7% during 40 days’ treatment [28]. Volumetric soil water content was measured with a Theta Probe soil moisture sensor (ML2; Delta-T Devices, U.K.).

### Measurements

Morphological and physiological parameters were determined during dry-down period. Leaf samples were harvested every 10 days for biochemical measurements. The samples were taken from the top of the canopy at 0 d (normal moisture, about 29% volumetric soil water content), 10 d (about 24% volumetric soil water content), 20 d (about 19% volumetric soil water content), 30 d (about 14% volumetric soil water content), 40 d (about 7% volumetric soil water content). The samples were frozen with liquid nitrogen and stored in sealed plastic bags at -80°C until analysis.

#### Turf quality, leaf relative water content, and leaf wilting

Turf quality (TQ) was classified based on a visual scale of 1–9, with 1 indicating yellow, dead leaves, 9 the best possible quality, and 6 the minimum acceptable turf quality according to DaCosta and Huang [29]. Leaf relative water content (RWC) was measured from fresh weight (FW) (≈ 0.1g), dry weight (DW) and turgid weight (TW) using the formula RWC (%) = [(FW—DW) / (TW—DW)] × 100. Leaves were collected from grasses and immediately weighed for measurement of FW. Samples were then immersed in deionized water and kept in the dark for 12 h at 4°C. After this, leaves were blotted dry and weighed immediately to determine TW. Next, samples were dried in an oven set to 80°C for at least 72 h and weighed for DW [30]. Leaf wilting rate was graded based on a visual scale of 0 to 100%, with 100% indicating complete, permanent wilting of the canopy [31].

#### Leaf electrolyte leakage and malondialdehyde (MDA) content

For electrolyte leakage (EL) measurement, fresh leaves (0.1 g) were collected and immediately rinsed with deionized water, then soaked in 20 mL deionized water. The initial conductivity of the solution (C_i_) was determined using a conductivity meter (DDSJ_308A; Shanghai Precision and Scientific Inc., China) after the leaves were incubated in deionized water for 24 h on a shaker. Leaves were then killed by boiling water at 100°C for 30 min. The final conductivity of killed tissues (C_m_) was determined after samples were cooled down to room temperature. Electrolyte leakage was the result of C_i_ / C_m_ [32].

The MDA content was determined according to Heath and Packer [33]. One milliliter aliquot of supernatant of leaf extracts was added to 2 mL of a reaction solution containing 20% (v/v) trichloroacetic acid and 0.5% (v/v) thiobarbituric acid. The mixture was heated at 100°C for 30 min and then quickly cooled in an ice-water bath. After the mixture was centrifuged at 10,000 × g for 10 min, the absorbance of the supernatant was read at 532 nm and 600 nm. Absorbance at 600 nm was subtracted from that at 532 nm, and MDA content was calculated by means of an extinction coefficient of 155 mM^-1^·cm^-1^.

#### Reactive oxygen species content

The O_2_^-^ production rate was measured using the methods of Jiang and Zhang [34] with slight modifications. Frozen leaves (0.2 g) were homogenized in 1 mL of 50 mM Tris-HCl (pH 7.5) and centrifuged at 5000 × g for 10 min at 4°C. The reaction mixture (1 mL) contained 100 μL supernatant and 0.5 mM 3-bis (2-methoxy-4-nitro-5-sulfophenyl) -2H-tetrazolium-5-carboxanilide inner salt (XTT sodium salt) and 0.2 mM NADH. The reduction of XTT was recorded at 490 nm for 10 min. The background absorbance was corrected in the presence of 50 units SOD. The O_2_^-^ production rate was measured using an extinction coefficient of 21.6 mM^-1^·cm^-1^. The H_2_O_2_ content was measured as described by Bernt and Bergmeyer [35]. Leaves (0.2 g) were homogenized in 1.5 mL of 100 mM sodium phosphate buffer (pH 6.8), and extractions were then centrifuged at 15,000 × g for 5 min at 4°C. Then 0.17 mL of supernatant was added to 0.83 mL peroxidase reagent containing 83 mM sodium phosphate (pH 7.0), 0.005% (w/v) o-dianisidine, and 40 μg peroxidase·mL^-1^. The solution was incubated at 30°C for 10 min, and 0.17 mL of 1 M perchloric acid was added to stop the reaction. The absorbance was read at 436 nm. The H_2_O_2_ concentration was calculated though the standard curve with known concentration.

#### Antioxidant enzymes activities

The activity of superoxide dismutase (SOD) was determined according to the method of Giannopolitis and Ries [36]. Briefly, the assay medium contained 50 mM phosphate buffer (pH 7.8), 13 mM methionine, 75 mm p-nitro blue tetrazolium chloride (NBT), 2 mm riboflavin, 0.1 mM ethylene diaminetetraacetic acid (EDTA), and 20 to 50 mL enzyme extract. The changes of absorbance at 560 nm were measured with a spectrophotometer (UV-2802S, UNICO, Spain). One unit of enzyme activity was determined as the amount of enzyme required to inhibit the NBT reduction by 50%. The activity of guaiacol peroxidase (POD) was assayed by recording the increase in absorbance at 470 nm for 1 min. The reaction mixture contained 25 μL of 20 mM guaiacol, 1.42 mL of 10 mM phosphate buffer (pH 7.0), and 50 μL of enzyme extract. The reaction was started with the addition of 10 μL 40 mM H_2_O_2_ [37]. Catalase (CAT) activity was measured according to Chance and Maehly [38]. The reaction mixture contained 50 mM sodium phosphate buffer (pH 7.0), 45 mM H_2_O_2_ and 100 μL of extracted solution. The reaction was started by adding the enzyme solution. The decrease in absorbance at 240 nm was read every 10 s for 60 s. One unit CAT activity was determined as the absorbance change of 0.01 per minute. The activity of ascorbate peroxidase (APX) was determined a decrease in absorbance at 290 nm for 1 min. The 1.5 mL assay contained 50 mM potassium phosphate buffer (pH 7.0), 0.5 mM ascorbic acid, 0.1 mM EDTA, 0.1 mM H_2_O_2_, and 0.15 mL of enzyme. The reaction was initiated by adding H_2_O_2_ [38].

#### Isozymes discrimination

Antioxidant isozymes were analyzed on discontinuous polyacrylamide gel electrophoresis (PAGE) under nondenaturing, nonreducing conditions as described by Laemmli [39]. SOD was detected on 12% acrylamide gel, and POD, CAT, and APX were detected on 10% gel with equal amount of soluble protein per lane. Electrophoresis was carried out using a Bio-Rad mini-gel system at 4°C for 3–4 h in 0.04 mol Tris (hydroxymethyl) aminomethane-glycine buffer (pH 8.3) for SOD, POD, CAT, and in the same buffer with addition of 2 mmol ascorbate for APX.

The SOD isozymes were visualized using the NBT staining method as described by Beauchamp and Fridovich [40]. Briefly, the gel was soaked in 100 mL of solution containing 50 mM sodium phosphate buffer (pH 7.8) and 2.5 mM NBT for 25 min in the dark at room temperature, and then was washed twice with sodium phosphate buffer (pH 7.8) followed by soaking gel in 28 μM riboflavin and 28 mM tetramethylethylenediamine (TEMED) in 36 mM sodium phosphate buffer (pH 7.8) for 40 min. Finally, gel was soaked in 50 mM sodium phosphate buffer (pH 7.8) containing 0.1 mM EDTA with gentle agitation and exposed to incandescent lights (40 μmol·m^-2^·s^-1^) until the transparent bands (SOD isozymes) were visible. For the staining of POD isoforms, the gel was incubated in a sodium phosphate solution (10 mM sodium phosphate and 150 mM sodium chloride, pH 6.0) for 45 min. After being washed with 100 mM potassium phosphate buffer (pH 6.4), the gel was stained in 100 mM potassium phosphate buffer (pH 6.4) containing 20 mM guaiacol and 5.55 mM H_2_O_2_ for 5–10 min until the bands (POD isozymes) were visible [41]. The CAT isozyme staining was visualized according to Woodbury et al. [42]. The gel was washed three times with deionized water to remove the buffer from the gel surface where staining occurred. The gel was incubated in 0.3% H_2_O_2_ for 10 min at room temperature in darkness with light agitation, and then incubated in the stain solution containing 1% (w/v) FeCl_3_ and 1% (w/v) K_3_Fe(CN)_6_ in deionized water under room temperature for 3–5 min or until the light yellow bands (CAT isozymes) were clearly visible. All the steps of detection of APX isozymes in the gel was performed according to the procedure described in López-Huertas et al. [43]. After the electrophoretic separation, the gel was incubated in 50 mM sodium phosphate buffer (pH 7.0) containing 4 mM ascorbate and 2 mM H_2_O_2_ in darkness with light agitation for 20 min. The gel was then washed with sodium phosphate buffer (pH 7.0) briefly and submerged in 50 mM sodium phosphate buffer (pH 7.8) containing 28 mM TEMED and 1.25 mM NBT in the dark for 10 min. The APX isozymes were observed as the achromatic bands.

#### Nitrate reductase activity

The activity of leaf nitrate reductase activity was determined using the methods of Chanda [44]. Briefly, about 0.2 g fresh leaf tissue was cut into 0.5 cm lengths. Then, the leaf sections were immersed in 15 mL of 0.05 M potassium phosphate buffer (pH 7.8) with 1% n-propanol and 50 mM KNO_3_. The samples were vacuum infiltrated for 10 min to ensure infiltration of incubation buffer and then incubated in darkness for 1 h at 30°C. Next, 1 mL solution was transferred to 10 mL culture tubes. The nitrite formed was estimated colorimetrically by adding 750 μL of 1% sulfanilamid in 3 M HCl, and 750 μL of 0.02% N-naphthyl-ethylenediamine hydrochloride. Absorption was measured at 540 nm. For each run, blanks and four nitrite standards (1, 5, 10, and 25 μM KNO_2_) were included.

#### Experimental design and statistical analysis

The experiment was a randomized complete block design with four replications. There were two factors (CK and N) with CK at three levels and N at two levels. Irrigation conditions were not deemed treatments, but environmental stresses to which the grasses were uniformly suffered so as to test the effects of the N and CK treatments. All statistical analyses were performed using SPSS (SPSS10.0, Chicago IL, USA). The means were calculated with four biological repeats and analyzed with least significant difference (LSD) test at a probability level of 0.05, except as otherwise stated herein.

## Results

### Turf quality, leaf relative water content, and leaf wilting

Drought stress reduced TQ, but CK and high N improved TQ under both drought stress and well-watered conditions. Under drought stress condition, TQ of the bentgrass treated with 10 μM and 100 μM CK at 30 d and 40 d were higher than that without CK treatment under both N condition s. Grasses treated with high N exhibited higher TQ than that in low N since 20 d, and the increment rate reach at 7% at 40 d when averaged across CK levels (Table 1).

**Table 1:** Cytokinin and nitrogen impact on turf quality and leaf relative water content of creeping bentgrass subjected to two moisture regimes.

Moisture^§^	Nitrogen	Cytokinin	0 d	10 d	20 d	30 d	40 d
			**Turf quality**				
			**(1–9; 9 = ideal green; healthy turfgrass)**				
**Well-watered**	**low N**	**0**	7.6aA^‡^	7.7bcA	7.6bA	7.7dA	7.6dA
		**10**	7.7aA	7.7abcA	7.7bA	7.8cA	7.7cdA
		**100**	7.7aB	7.8aAB	7.8aAB	7.9cA	7.9abcA
**Well-watered**	**high N**	**0**	7.6aD	7.7bcCD	7.7bC	8.1aA	7.8bcdB
		**10**	7.7aC	7.7bcC	7.9aB	8.0bA	7.9abAB
		**100**	7.7aC	7.8abC	7.9aB	8.1aA	8.0aA
**Drought stress**	**low N**	**0**	7.6aA	7.6cA	7.3dB	6.4iC	5.0iD
		**10**	7.6aA	7.6cA	7.4cB	7.1gC	5.3hD
		**100**	7.7aA	7.7abcA	7.5cB	7.2fC	5.8fD
**Drought stress**	**high N**	**0**	7.6aAB	7.6cA	7.5cB	6.8hC	5.2hiD
		**10**	7.7aA	7.7bcA	7.5cB	7.3fC	5.6gD
		**100**	7.7aA	7.7abcA	7.6bA	7.4eB	6.4eC
			**Leaf relative water content**				
			**(%)**				
**Well-watered**	**low N**	**0**	89.9aA	89.9bcdA	87.9dB	87.8dB	86.8dC
		**10**	90.6aA	90.3bcdAB	88.1dC	88.9cBC	88.4cdC
		**100**	91.6aA	91.5abA	90.7bA	91.0bA	90.1bcA
**Well-watered**	**high N**	**0**	90.4aB	92.8aA	88.8cdC	89.4cBC	89.4bcBC
		**10**	91.6aA	91.2bcA	90.9bA	91.8bA	90.7abA
		**100**	90.8aB	91.4abcAB	93.4aA	93.7aA	92.6aA
**Drought stress**	**low N**	**0**	91.1aA	88.7dB	83.6fC	76.5hD	66.1iE
		**10**	90.0aA	89.2dA	86.7eB	79.2gC	71.5ghD
		**100**	91.1aA	90.3bcdA	88.1dB	81.2fC	74.7fD
**Drought stress**	**high N**	**0**	91.7aA	89.7cdA	86.0eB	79.3gC	69.7hD
		**10**	91.3aA	89.8bcdB	86.2eC	81.2fD	73.3fgE
		**100**	91.7aA	90.4bcdAB	89.8bcB	83.1eC	77.3eD

§: The volumetric soil water content was maintained at about 29% for well-watered regime; For drought stress regime, the volumetric soil water content decreased from 29% to 7% during 40 days drought treatment

‡: Means with same lowercase letters within each column or uppercase letters within each row of each data set are not significantly different at P< 0.05.

Leaf RWC decreased during drought stress, while grasses treated with CK and high N showed higher leaf RWC than no CK and low N treatment under both water conditions. Leaf RWC in all treatments was maintained at about 90% under well-watered condition. During the drought period, high N treated grasses showed higher leaf RWC than low N treated grasses. And compared with no CK spray, the treatments with 10 μM CK and 100 μM CK in low N plants enhanced leaf RWC ratings by 8% and 13%, while 5% and 10% in high N condition, respectively, as observed on the last day (Table 1).

**Fig 1:**
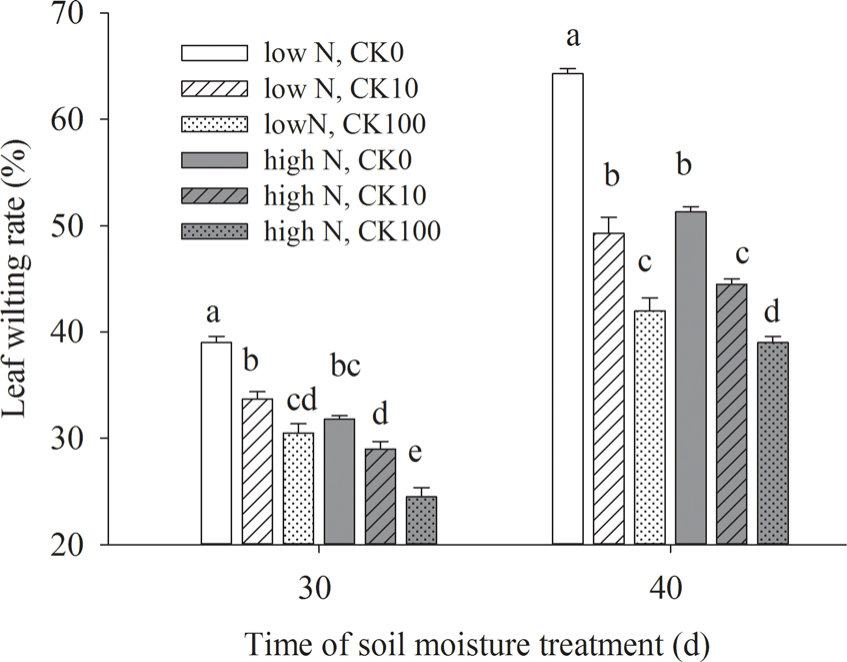
Effects of cytokinin *trans*-zeatin riboside (CK) and nitrogen on leaf wilting rate of creeping bentgrass under drought stress. Different lowercase letter means significant difference (P < 0.05) between treatments on the same day.

Drought stress led to a significant increase in leaf wilting rate, but CK and high N treatments delayed leaf wilting. At the last day of water stress, leaf wilting rate in low N grasses treated with 10 μM CK and 100 μM CK were 30% and 40% lower than that without CK treatment in low N condition, respectively. Similarly, grasses under high N conduction treated with 10 μM and 100 μM CK presented significantly lower leaf wilting rate than non-CK treatment. Moreover, High N grasses showed significantly lower leaf wilting rate than low N grasses under drought stress condition (Fig 1).

### Leaf electrolyte leakage and malondialdehyde (MDA) content

Drought stress significantly increased EL, but CK and high N treatments reduced EL as measured during the water stress. At the last day of water stress, EL of the grasses treated with 10 μM CK and 100 μM CK were 11% and 21%, respectively, lower than that without CK treatment when averaged across N condition. The EL was 8% greater in low N plants versus in high N plants (averaged across CK levels) under drought stress condition (Table 2).

**Table 2:** Cytokinin and nitrogen impact on leaf electrolyte leakage (EL) and malondialdehyde (MDA) content of creeping bentgrass subjected to two moisture regimes.

Moisture^§^	Nitrogen	Cytokinin	0 d	10 d	20 d	30 d	40 d
			**EL**				
			**(%)**				
**Well-watered**	**low N**	**0**	21.0aB^‡^	22.9abAB	23.9dA	23.8gA	24.4fA
		**10**	21.7aAB	21.6abcAB	21.2eB	22.7ghA	22.4fghAB
		**100**	21.6aAB	20.1cB	21.1eB	22.9ghA	21.5ghiAB
**Well-watered**	**high N**	**0**	21.1aC	22.9abAB	21.8eBC	22.1ghBC	23.6fgA
		**10**	22.4aA	22.7abA	20.5efB	21.5hiAB	21.1hiAB
		**100**	22.5aA	21.5abcA	19.4fB	19.7iB	19.3iB
**Drought stress**	**low N**	**0**	21.0aD	23.0abD	32.5aC	53.8aB	62.9aA
		**10**	21.2aD	22.3abD	30.9bC	46.3cB	57.1cA
		**100**	21.8aD	21.9abD	26.8cC	40.7eB	52.9dA
**Drought stress**	**high N**	**0**	21.5aD	23.3aD	30.9bC	49.5bB	59.5bA
		**10**	21.0aD	21.8abD	28.3cC	44.0dB	53.2dA
		**100**	21.5aD	21.3bcD	25.0dC	38.4fB	48.1eA
			**MDA**				
			**(n mol·g**^**-1**^**)**				
**Well-watered**	**low N**	**0**	34.1aD	37.7aC	38.2eBC	39.9fAB	41.0fA
		**10**	35.5aC	36.2abBC	37.9eAB	38.1fAB	38.3fgA
		**100**	35.5aAB	35.1bB	37.0eAB	37.1fAB	37.5fgA
**Well-watered**	**high N**	**0**	34.4aB	37.5aA	37.5eA	38.3fA	39.3fgA
		**10**	35.3aA	36.1abA	36.6eA	37.8fA	36.9fgA
		**100**	35.2aA	35.0bA	36.4eA	35.5fA	36.1gA
**Drought stress**	**low N**	**0**	35.1aD	37.8aD	79.5aC	95.6aB	115.2aA
		**10**	34.6aD	36.4abD	68.4bC	80.5bcB	106.8bA
		**100**	35.4aD	36.3abD	47.8dC	70.8dB	83.5dA
**Drought stress**	**high N**	**0**	35.5aD	37.8aD	70.5bC	86.1bB	104.1bA
		**10**	35.9aD	38.1aD	51.2cC	76.8cdB	91.2cA
		**100**	35.9aD	34.9bD	46.2dC	54.9eB	71.5eA

§: The volumetric soil water content was maintained at about 29% for well-watered regime; For drought stress regime, the volumetric soil water content decreased from 29% to 7% during 40 days drought treatment

‡: Means with same lowercase letters within each column or uppercase letters within each row of each data set are not significantly different at P< 0.05.

Leaf MDA contents in creeping bentgrass increased during drought stress, however, CK and high N treatments decreased MDA contents. Under drought stress and well-watered conditions, leaf MDA contents in high N treated plants were 15% and 4%, respectively, lower than those in low N treated plants at 40 d. When averaged across two N levels in the stressed group, plants treated with CK10 and CK100 showed 11% and 41%, respectively, lower than that with CK0 treatment (Table 2).

### Reactive oxygen species contents

ROS were quite stable in the well-watered plants but significantly increased during the water stress, and the H_2_O_2_ and O_2_^-^ contents tripled and doubled, respectively, in the treatment of low N without CK in the last day of stress period. High N and CK treatments inhibited the burst of ROS in the grasses under drought stress. High N treated plants had low H_2_O_2_ and O_2_^-^ concentrations, which were 9% and 11% (averaged across CK levels) lower than those in the low N plants at 40 d, respectively. Moreover, both CK10 and CK100 significantly reduced H_2_O_2_ and O_2_^-^ production for both N levels during the stress period (Table 3).

**Table 3:** Cytokinin and nitrogen impact on hydron peroxide (H_2_O_2_) concentration and superoxide radical (O2^-^) production rate of creeping bentgrass subjected to two moisture regimes.

Moisture^§^	Nitrogen	Cytokinin	0 d	10 d	20 d	30 d	40 d
			**H**_**2**_**O**_**2**_				
			**(μmol·g**^**-1**^**)**				
**Well-watered**	**low N**	**0**	22.7aB^‡^	23.3abB	24.9dA	25.0eA	25.1fA
		**10**	22.4abB	23.5abAB	23.8dA	23.8efA	23.0fgAB
		**100**	22.5abA	22.2abA	21.3eAB	21.7fgAB	20.7ghB
**Well-watered**	**high N**	**0**	22.2abB	23.5abA	24.2dA	21.9fgB	21.4ghB
		**10**	21.2bAB	22.8abA	21.4eAB	20.4gAB	20.1ghB
		**100**	21.1bAB	21.7bA	19.2fC	19.8gBC	18.8hC
**Drought stress**	**low N**	**0**	22.1abD	24.8aD	46.0aC	52.5aB	69.4aA
		**10**	22.7aD	23.6abD	35.1bC	41.6bB	49.2cA
		**100**	21.8abD	23.1abD	32.2cC	37.8cB	43.4dA
**Drought stress**	**high N**	**0**	22.6aD	23.6abD	36.1bC	43.6bB	62.6bA
		**10**	22.2abD	23.4abD	31.8cC	39.1cB	46.6cA
		**100**	22.0abD	22.1bD	25.8dC	30.1dB	39.9eA
			**O**_**2**_^**-**^				
			**(μmol·g**^**-1**^**·min**^**-1**^**)**				
**Well-watered**	**low N**	**0**	17.7aC	17.8aC	19.6dAB	20.0eA	18.7fBC
		**10**	17.2aB	17.5aB	18.0eAB	18.7fA	17.4ghB
		**100**	17.1aA	16.8abA	17.5eA	16.7gA	16.6hA
**Well-watered**	**high N**	**0**	17.1aC	17.6aBC	18.2eB	19.4efA	17.8fgBC
		**10**	17.2aA	17.1abA	17.7eA	17.1gA	16.7hA
		**100**	16.9aA	15.8cB	15.8fB	15.5hBC	15.0iC
**Drought stress**	**low N**	**0**	17.4aD	17.8aD	26.9aC	31.6aB	35.1aA
		**10**	17.2aD	17.8aD	21.2cC	25.3bB	27.5bcA
		**100**	17.2aD	17.3aD	20.0cdC	24.0cB	25.7dA
**Drought stress**	**high N**	**0**	16.9aE	17.6aD	24.3bC	26.4bB	28.0bA
		**10**	17.2aD	17.5aD	20.9cC	24.1cB	26.5cdA
		**100**	17.0aE	16.3bcD	18.2eC	22.5dB	24.6eA

§: The volumetric soil water content was maintained at about 29% for well-watered regime; For drought stress regime, the volumetric soil water content decreased from 29% to 7% during 40 days drought treatment

‡: Means with same lowercase letters within each column or uppercase letters within each row of each data set are not significantly different at P< 0.05.

### Antioxidant enzymes activities

**Fig 2:**
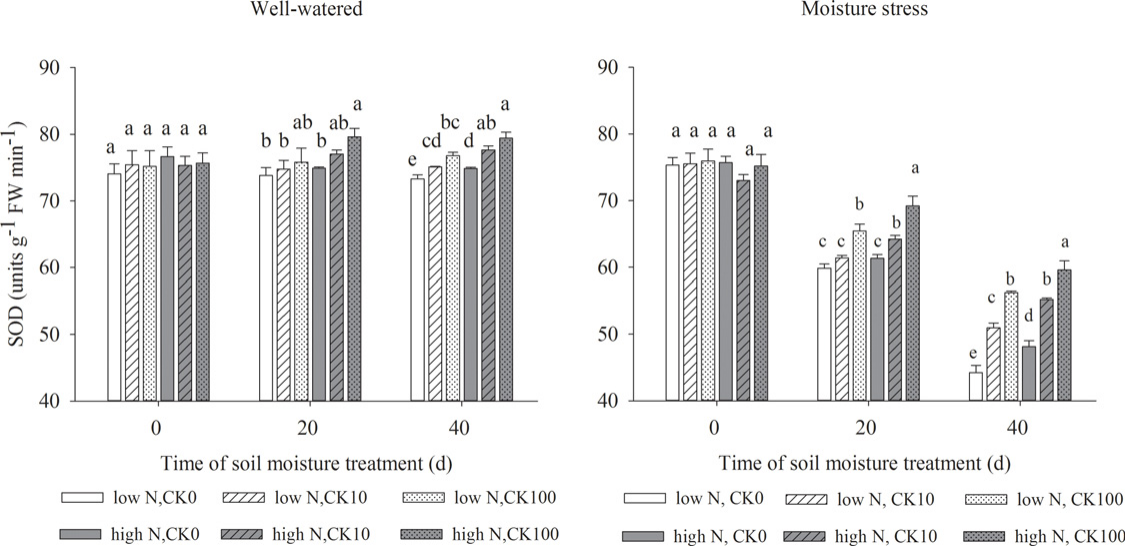
Activities of superoxide dismutase (SOD) in leaves of drought stressed creeping bentgrass as affected by cytokinin *trans*-zeatin riboside (CK) and nitrogen. Bars having different letter indicate no significant enzyme activity difference (P < 0.05) between treatments on the same day.

**Fig 3:**
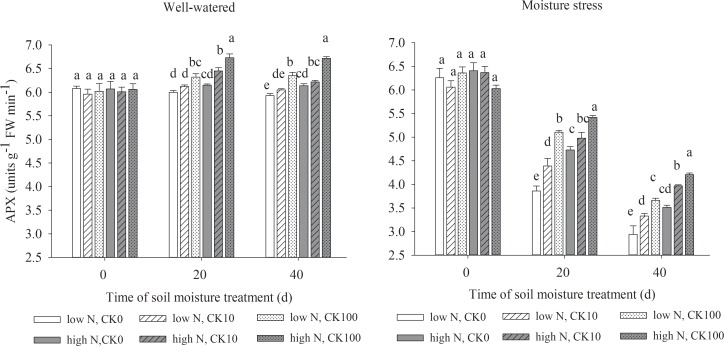
Activities of ascorbate peroxidase (APX) in leaves of drought stressed creeping bentgrass as affected by cytokinin *trans*-zeatin riboside (CK) and nitrogen. Bars having different letter indicate no significant enzyme activity difference (P < 0.05) between treatments on the same day.

**Fig 4:**
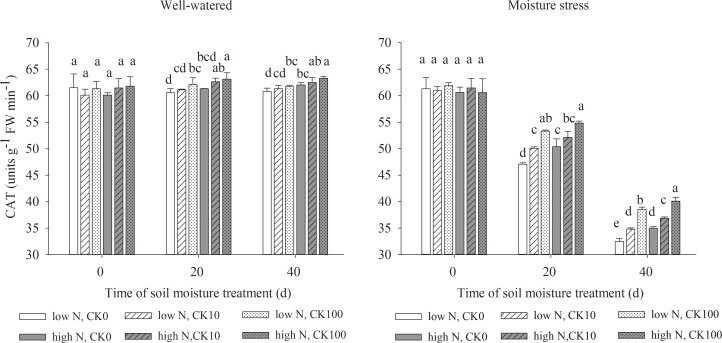
Activities of catalase (CAT) in leaves of drought stressed creeping bentgrass as affected by cytokinin *trans*-zeatin riboside (CK) and nitrogen. Bars having different letter indicate no significant enzyme activity difference (P < 0.05) between treatments on the same day.

**Fig 5:**
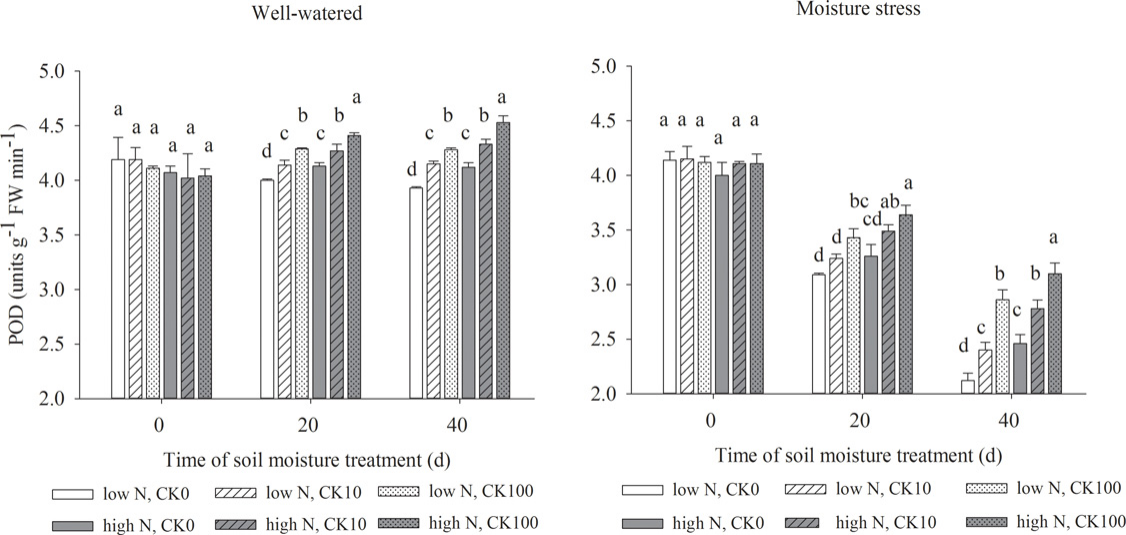
Activities of guaiacol peroxidase (POD) in leaves of drought stressed creeping bentgrass as affected by cytokinin and nitrogen. Bars having different letter indicate no significant enzyme activity difference (P < 0.05) between treatments on the same day.

Drought stress led to significant declines in the activities of SOD, APX, CAT and POD, but both CK and N treatments showed the positive effects to maintain the enzyme activities under both water regimes. Under well-watered condition, there were significantly higher activities of SOD, APX, POD and CAT in CK100 treated grasses and higher activities of POD in CK10 treated grasses compared with those without CK treatments. Under water stress, both CK10 and CK100 treatments dramatically increased the activities of SOD, APX, CAT and POD, respectively. Moreover, high N treatment increased the activities of four antioxidant enzymes in grasses under both soil moisture regimes (Figs 2–5).

### Isozymes discrimination

**Fig 6:**
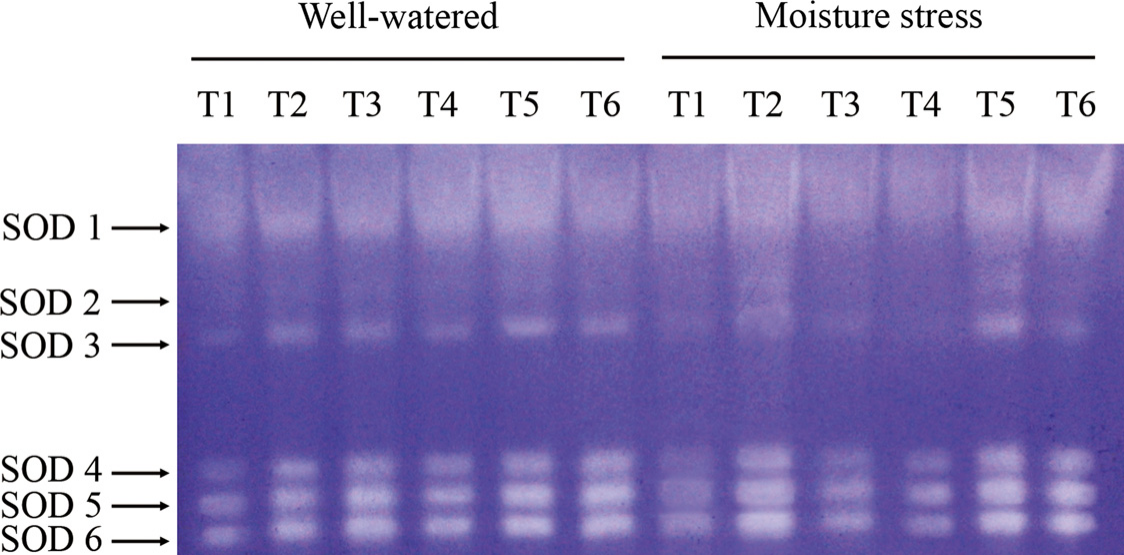
Native gel stained for the activity of SOD isoforms of drought stressed creeping bentgrass treated with cytokinin *trans*-zeatin riboside (CK) and nitrogen. T1 to T6 represent low N + CK0, low N + CK100, low N + CK10, high N + CK0, high N + CK100, high N + CK10 of creeping bentgrass at 40 d, respectively.

Six isoforms of SOD were detected by the native PAGE gel of creeping bentgrass under all treatments (assigned as SOD1 to SOD 6 according to their migration rates). There was no apparent SOD isoform pattern or relative stain intensity difference between N treatments under both moisture regimes, but SOD in the grasses treated with 10 μM and 100 μM CK showed stronger stain intensity than the grasses treated without CK. However, the differences among CK treated plants were less remarkable in well-watered plants (Fig 6).

**Fig 7:**
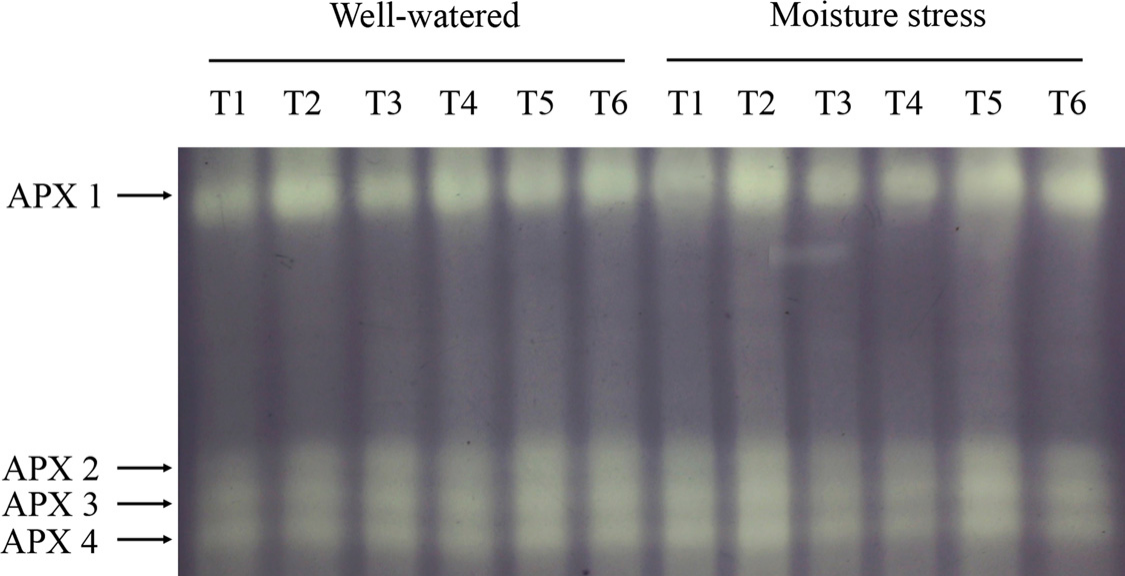
Native gel stained for the activity of APX isoforms of drought stressed creeping bentgrass treated with cytokinin *trans*-zeatin riboside (CK) and nitrogen. T1 to T6 represent low N + CK0, low N + CK100, low N + CK10, high N + CK0, high N + CK100, high N + CK10 of creeping bentgrass at 40 d, respectively.

Four isoforms of APX were revealed and arbitrarily assigned as APX1 to APX4. There was also no apparent APX isoform pattern or relative stain intensity difference between N treatments under both moisture regimes. Under soil moisture stress, the stain intensity of APX1 in the grasses treated with 10μM CK and 100 μM CK were stronger than those without CK treatment, regardless of N levels. Moreover, APX2 to 4 in 100 μM CK treated grasses also showed higher the stain intensity (Fig 7).

**Fig 8:**
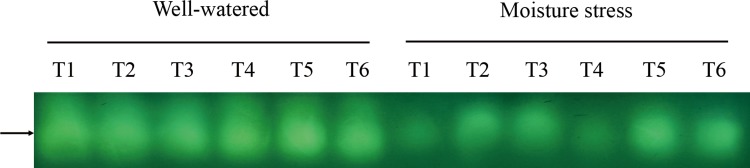
Native gel stained for the activity of CAT isoform of drought stressed creeping bentgrass treated with cytokinin *trans*-zeatin riboside (CK) and nitrogen. T1 to T6 represent low N + CK0, low N + CK100, low N + CK10, high N + CK0, high N + CK100, high N + CK10 of creeping bentgrass at 40 d, respectively.

Only one isoform of CAT was identified, and drought stressed plants showed relatively lower activity than the plants under well-watered condition. With soil moisture stress, high N plants showed stronger stain intensity than low N plants. And the grasses treated with 10 μM CK and 100 μM CK showed higher stain intensity than the grasses without CK treatment. There was no apparent CAT isoform pattern or relative stain intensity difference between well-watered plants (Fig 8).

**Fig 9:**
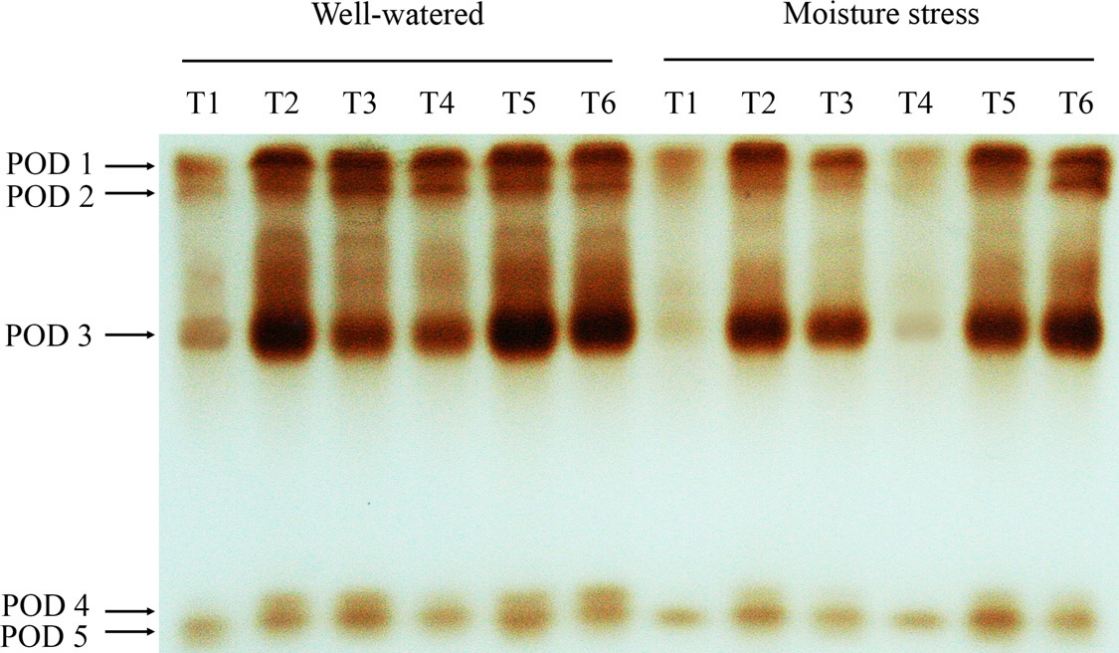
Native gel stained for the activity of POD isoforms of drought stressed creeping bentgrass treated with cytokinin *trans*-zeatin riboside (CK) and nitrogen. T1 to T6 represent low N + CK0, low N + CK100, low N + CK10, high N + CK0, high N + CK100, high N + CK10 of creeping bentgrass at 40 d, respectively.

Five POD isozymes were detected and the drought stress significantly reduced the activities of isoforms. Under soil moisture stress, the bands intensity of CK10 and CK100 treated plants were stronger than the grasses treated with CK0, respectively. Plants treated with high N exhibited a stronger intensity of POD isoforms than that with low N (Fig 9).

### Nitrate reductase (NRA) activity

Drought stress led to significant reduction in NRA, and high N treated grasses showed higher NRA under both moisture conditions. Under drought stress condition, NRA in the bentgrass treated with 10 μM CK and 100 μM CK were 4% and 8%, respectively, higher than that without CK treatment at 40 d when averaged across N levels. However, only 100 μM CK treatment enhanced NRA in well-watered plants compared with CK0 treatment. Plants receiving high N treatment showed 7% (averaged across CK levels) higher NRA than that with low N (Table 4).

**Table 4:** Cytokinin and nitrogen impact on leaf nitrate reducrase (NRA) activity of creeping bentgrass subjected to two moisture regimes.

Moisture^§^	Nitrogen	Cytokinin	0 d	10 d	20 d	30 d	40 d
			Leaf NRA activity				
			(μg NO_2_^-^·g^-1^ FW·h^-1^)				
**Well-watered**	**low N**	**0**	20.2aA^‡^	20.1bA	20.4bA	19.4eB	19.5dB
		**10**	20.0aB	20.5abAB	21.0bA	20.1deB	20.2cdB
		**100**	20.0aC	20.1abBC	21.3bAB	21.4bcA	21.0bABC
**Well-watered**	**high N**	**0**	20.1aB	20.5abAB	21.4bA	20.4cdAB	20.8bcAB
		**10**	20.0aC	20.8abBC	22.8aA	21.8abAB	21.5bABC
		**100**	20.0aC	21.1aB	23.7aA	22.7aA	22.6aA
**Drought stress**	**low N**	**0**	20.5aA	20.0bA	17.3eB	15.9hC	14.0hD
		**10**	20.0aA	20.0bA	17.5deB	16.5ghC	14.6ghD
		**100**	20.4aA	20.2abA	18.4cdB	17.1fgC	15.3fgD
**Drought stress**	**high N**	**0**	20.1aA	20.2abA	17.6deB	16.5fhC	15.0fgD
		**10**	20.0aA	20.0bA	18.4cdB	17.1fgC	15.6efD
		**100**	20.5aA	20.3abA	19.2cB	17.7fC	16.1eD

§: The volumetric soil water content was maintained at about 29% for well-watered regime; For drought stress regime, the volumetric soil water content decreased from 29% to 7% during 40 days drought treatment

‡: Means with same lowercase letters within each column or uppercase letters within each row of each data set are not significantly different at P< 0.05.

## Discussion

In our study, both CK10 and CK100 applications increased TQ, leaf RWC, while reduced leaf wilting, EL, and MDA contents at the same N level, particularly under drought stress. These results are consistent with many previous studies with creeping bentgrass [10,14,22]. MDA content is an indicator for assessing the stability of cell membrane [5]. The alleviating effects of CK on the stress injury of creeping bentgrass could be due to the protective effects of the cytokinin on cell membranes. Liu et al. [45] noted that CK at 1 to 10 μM increased turfgrass performance and heat stress tolerance of creeping bentgrass. Our previous study by co-author Zhang indicated that the CK at both 10 uM and 100 uM are effective in promoting creeping bentgrass tolerance to heat stress (personal communication). In the study herein, we used CK concentrations of 10 and 100 μM. It appears CK at both concentrations improved creeping bentgrass tolerance to drought stress. Our results showed that high N increased TQ and leaf RWC, reduced leaf wilting rate of creeping bentgrass at all CK treatments under drought stress. Similar results were found in drought stressed *Sophora davidii* [46] and heat stressed creeping bentgrass [21]. High N treated bentgrass showed lower EL and MDA contents than low N treatment at the same CK rate. Similar results were reported by Wang et al. [22] and Xu et al. [15]. High N treatment also has been reported to help maintain greater photosynthesis and photosynthetic N-use efficiency in plants [17]. These are the reasons to explain why high N treated grasses in our study had higher TQ and leaf RWC, and lower leaf wilting rate.

Our results showed that both CK10 and CK100 treatments, at the same N level, significantly enhanced leaf antioxidant enzyme activities, reduced H_2_O_2_ concentration and O_2_^-^ production rate of creeping bentgrass under drought stress. CK100 treatment also decreased ROS production under well-watered condition. Previous studies suggested that exogenous application of CK may alleviate ROS damage in various plants [14,47,48]. Drought stress induced excess and toxic ROS in cells, which may cause damage to cell membranes. Various antioxidant enzymes can scavenge ROS and protect cells under different abiotic stresses [49,50]. Our results suggested that exogenous CK could enhance the activities of antioxidant enzymes, and promote the antioxidant defenses against drought stress in creeping bentgrass. It was observed that zeatin riboside [51] or dihydrozeatin [52] treatment enhanced activities of CAT and SOD. Our data also showed high N treatment had dramatic effects on the reduction of toxic ROS and the increases of leaf antioxidant enzyme activities at all CK treatments. In previous studies, the activities of these four antioxidant enzymes increased concomitantly with a decrease of ROS production and MDA contents, or vice versa [7,22,26]. The results of our study showed that the antioxidant enzyme activities were higher at high N relative low N rate under 10 or 100 uM CK level and drought stress conditions (Figs 2, 3, 4 and 5), concomitant with declined MDA contents (Table 2). This indicated that high N may improve antioxidant metabolism and tolerance to drought tolerance by suppressing toxic ROS in creeping bentgrass.

It is well-known that enzyme isoforms may function differently due to their distinct properties, which could be more sensitive and subtler for plants in response to drought stress compared to their activities changes [53]. Our results indicated that the isozyme activities of all three enzymes except APX in the stressed plants showed a decline trend after prolonged drought stress. Previous studies reported that environment stress may enhance the expression level of isozymes, especially in the early or mid phases of the abiotic stresses [54]. However, Pan et al. [50] found that liquorice seedlings (*Glycyrrhiza uralensis* Fisch) showed lower SOD isoforms activity after a prolonged drought stress which is very similar to our study herein. Moreover, Hu et al. [55] found that the isozymes of SOD, POD and APX gradually declined for perennial ryegrass after 12 days salinity stress. Therefore, the different changes of isozyme activities might be related to species, stress type and its suffering time.

In our study, there was no apparent evidence of a difference in SOD isoform profiles between N treatments at the same CK rate. Medici et al. [56] did not observe any SOD isozymes regulated by N supply in plants. Wang et al. [22] did not found any evidence of different SOD isoform profiles in root or shoot between nitrogen treated grasses, and they also reported that no effects of cytokinn on SOD isoforms pattern, which were inconsistent with our results. CK10 and CK100 treatments in our study dramatically enhanced SOD isoforms activities, especially SOD4 to SOD6. Previous report on SOD isoforms of creeping bentgrass suggested that the three bands were most likely Cu/Zn-SOD isoforms [49]. The abundant Cu/Zn-SOD isoforms were found in the cytosol and chloroplasts, and they were identified as main enzymes to avoid negative effects of ROS [7]. Our results suggested that these isomers were responsible for the observed activity increase under CK10 and CK100 treatments. In this study, four APX isozymes were detected in creeping bentgrass in response to drought stress. However, Wang et al. [22] reported only one band of APX isoform was observed in both roots and shoots of heat stressed creeping bentgrass. Six APX isozymes were detected in rice (*Oryza sativa* L.) in response to drought stress [57]. These different results suggest that the amount of APX isoforms observed are species and stress types dependent. Like SOD, native gel did not show an obvious effect of nitrogen but did show a significant effect of CK on APX isoforms stain intensity. APX1 was enhanced by CK100 treatment in both moisture regimes, which corresponded to the increase in APX activity. Only one band of CAT isoform was observed in our study, which is consistent with what He and Huang [54] reported in Kentucky bluegrass (*Poa pratensis* L.). Our data indicate that high N treated plants showed a slight increase in CAT isoform activity at all CK applications under both moisture conditions. The result is well in agreement with that of Medici et al. [56], who reported that the application of increased N slightly increased the CAT isoform activity. Our results indicated that both CK10 and CK100 showed a significant effect on CAT isoform stain intensity, which was consistent with the CAT activity assay. The data suggest that this isomer was responsible for the observed activity increase under CK and high N treatments. The lower intensity of CAT isoform in drought stress condition indicated that CAT metabolism may be more vulnerable to the persecution of the stress. For POD, five isoforms were detected in creeping bentgrass. Nitrogen treatment, in our study, had effects on POD isozymes under drought stress, and high N induced stronger intensity for POD1 and POD3. A previous report on POD isozymes of astragalus membranaceus (*Fabaceae*) suggested high N application level did enhance the activities [58]. The change in the staining intensities showed a similar trend compared to the quantitative changes of the POD activities. In addition, our results showed CK10 and CK100 treatments also induced increases of the POD isozymes. Previous study reported that CK could enhance the activities of isozymes, thus reduced the level of ROS in the delayed-senescence wheat leaves [11]. Our results suggested the increased isozymes activities, which resulted from exogenous CK and nitrogen, could contribute to the enhanced antioxidant mechanism of creeping bentgrass resisting drought stress.

Our results also showed that the positive influence of CK and nitrogen treatments on NRA activity. Higher NRA was detected under high N treated plants. Exogenous CK10 and CK100 also showed induction effects on NRA at both N levels. Similar effects of N and CK on NRA were observed in creeping bentgrass by Wang et al. [59]. The positive effects suggest that CK and N applications can also improve N assimilation, particularly under drought stress conditions. Thus the changes in NRA in our study were consistent with antioxidant enzymes and isozymes.

## Conclusion

In summary, both CK and nitrogen had the effects on all measured parameters in creeping bentgrass. Application of CK and high N promoted creeping bentgrass visual quality and enhanced N metabolism, especially under drought stress conditions. Exogenous CK and nitrogen increased the activities of NRA, activity of antioxidant enzymes and expression of their isozymes under drought stress. The results of this study indicated that CK or nitrogen may improve drought tolerance of creeping bentgrass and protect cell membranes from oxidative damage under drought stress by promoting antioxidant metabolism and N metabolism. The results of our study suggest that biweekly foliar application of 10 or 100 μM *trans*-zeatin riboside and 7.5 kg N·ha^-1^ may be suitable rates to improve the plant growth and visual quality under drought stress environment in creeping bentgrass. Further research is warranted to identify the best CK rate for improving drought stress tolerance in creeping bentgrass.

## Abbreviations

APX: ascorbate peroxidase
CAT: catalase
CK: cytokinin
DW: dry weight
EDTA: ethylene diaminetetraacetic acid
EL: electrolyte leakage
ET: Evaportranspiration
FW: fresh weight
LSD: least significant difference
LWR: leaf wilting rate
MDA: malondialdehyde
N: nitrogen
NBT: p-nitro blue tetrazolium chloride
NRA: nitrate reductase activity
PAGE: polyacrylamide gel electrophoresis
POD: guaiacol peroxidase
ROS: reactive oxygen species
RWC: relative water content
SOD: superoxide dismutase
TEMED: tetramethylethylenediamine
TQ: turf quality
TW: turgid weight
XTT: 3-bis (2-methoxy-4-nitro-5-sulfophenyl) -2H-tetrazolium-5-carboxanilide inner salt
